# Use of NanoBiT and NanoBRET to characterise interleukin‐23 receptor dimer formation in living cells

**DOI:** 10.1111/bph.16018

**Published:** 2023-01-10

**Authors:** Charles S. Lay, Laura E. Kilpatrick, Peter D. Craggs, Stephen J. Hill

**Affiliations:** ^1^ Division of Physiology, Pharmacology and Neuroscience, School of Life Sciences University of Nottingham Nottingham UK; ^2^ Centre of Membrane Proteins and Receptors University of Birmingham and Nottingham The Midlands UK; ^3^ Medicine Design, Medicinal Science and Technology GlaxoSmithKline Stevenage UK; ^4^ Division of Bimolecular Science and Medicinal Chemistry, School of Pharmacy, Biodiscovery Institute University of Nottingham Nottingham UK; ^5^ Crick‐GSK Biomedical Linklabs GlaxoSmithKline Stevenage UK

**Keywords:** cytokine‐receptor, IL12Rβ1, IL23R, Interleukin‐23, NanoBiT, NanoBRET, STAT3

## Abstract

**Background and Purpose:**

Interleukin‐23 (IL‐23) and its receptor are important drug targets for the treatment of auto‐inflammatory diseases. IL‐23 binds to a receptor complex composed of two single transmembrane spanning proteins IL23R and IL12Rβ1. In this study, we aimed to gain further understanding of how ligand binding induces signalling of IL‐23 receptor complexes using the proximity‐based techniques of NanoLuc Binary Technology (NanoBiT) and Bioluminescence Resonance Energy Transfer (BRET).

**Experimental Approach:**

To monitor the formation of IL‐23 receptor complexes, we developed a split luciferase (NanoBiT) assay whereby heteromerisation of receptor subunits can be measured through luminescence. The affinity of NanoBiT complemented complexes for IL‐23 was measured using NanoBRET, and cytokine‐induced signal transduction was measured using a phospho‐STAT3 AlphaLISA assay.

**Key Results:**

NanoBiT measurements demonstrated that IL‐23 receptor complexes formed to an equal degree in the presence and absence of ligand. NanoBRET measurements confirmed that these complexes bound IL‐23 with a picomolar binding affinity. Measurement of STAT3 phosphorylation demonstrated that pre‐formed IL‐23 receptor complexes induced signalling following ligand binding. It was also demonstrated that synthetic ligand‐independent signalling could be induced by high affinity (HiBit) but not low affinity (SmBit) NanoBiT crosslinking of the receptor *N‐*terminal domains.

**Conclusions and Implications:**

These results indicate that receptor complexes form prior to ligand binding and are not sufficient to induce signalling alone. Our findings indicate that IL‐23 induces a conformational change in heteromeric receptor complexes, to enable signal transduction. These observations have direct implications for drug discovery efforts to target the IL‐23 receptor.

## Graphical abstract

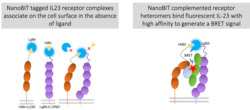

**Abbreviations**
BRET: Bioluminescence Resonance Energy Transfer
IL‐23R: interleukin‐23 receptor
NanoBiT: NanoLuc Binary Technology
NanoBRET: NanoLuc BRET
NL: NanoLuc

**What is already known**

- IL‐23 is a pro‐inflammatory cytokine involved in pathogen defence and the development of autoimmune diseases.
- IL‐23 mediates its effect through a heteromeric receptor complex composed of IL23R and IL12Rβ1.

**What does this study add**

- Inactive IL‐23 receptor complexes can form prior to cytokine engagement.
- High affinity constraint of the receptor *N*‐terminal domains can synthetically induce ligand‐independent signalling.

**What is the clinical significance**

- The interleukin‐23 receptor is an emerging drug target for the treatment of auto‐inflammatory diseases.
- These observations have direct implications for drug discovery efforts to target the IL‐23 receptor complex.

## 1 INTRODUCTION

The interleukin‐23 (IL‐23) cytokine is an important pro‐inflammatory mediator involved in host defence against bacterial and fungal pathogens (Kagami et al., 2010; Verreck et al., 2004). The production of IL‐23 is involved in the differentiation, expansion and maintenance of several pro‐inflammatory cell types, including T helper 17 (Th17), natural killer T (NKT), γδT and innate lymphoid cells (ILC) (Gaffen et al., 2014). In contrast to the protective effects of IL‐23 in infection, the cytokine has been shown to be a key mediator of auto‐inflammatory conditions including Crohn's disease and plaque psioriasis (Tang et al., 2012). Several anti‐IL‐23 biological therapies have been approved for the treatment of auto‐inflammatory conditions, and a new generation of peptide IL‐23 receptor antagonists is in clinical trials (Cheng et al., 2019; Chyuan & Lai, 2020). In addition to auto‐inflammatory indications, the IL‐23 pathway has been implicated in cardiovascular disease (Ye et al., 2020), cancer (Mirlekar & Pylayeva‐Gupta, 2021) and pain (Luo et al., 2021).

IL‐23 is a heterodimeric disulfide linked cytokine, belonging to the promiscuously pairing Interleukin‐12 (IL‐12) family, which comprises IL‐12, IL‐23, IL‐27 and IL‐35 (with IL‐39 reported in mice; Tait Wojno et al., 2019; Wang et al., 2016). The two components of IL‐23 are termed IL23p19 and IL12p40, the latter of which also forms half of the IL‐12 cytokine (Oppmann et al., 2000). The IL‐23 receptor is formed of two single transmembrane domain containing cytokine receptor chains, IL23R and IL12Rβ1, with IL12Rβ1 also forming half of the IL‐12 receptor (Parham et al., 2002). It has been demonstrated through mutagenesis and X‐ray crystallography that the IL23R specifically binds to IL23p19 and the IL12Rβ1 to IL12p40 (Bloch et al., 2018; Glassman et al., 2021; Schroder et al., 2015).

The binding of IL‐23 to its receptor leads to the transphosphorylation and activation of janus kinase (JAK) proteins, which are constitutively associated to sites in the intracellular domains of the receptor chains. JAK2 specifically binds to IL23R and TYK2 to IL12Rβ1 (Parham et al., 2002). Once the JAK proteins are activated, further signal transduction is mediated through phosphorylation of signalling proteins including dimeric members of the signal transducer and activator of transcription (STAT) family, with STAT3 dimers being one of the most prominent transducers (Floss et al., 2013; Parham et al., 2002).

The results of IL‐23 stimulation on cell fate have been well studied; however, the mechanism by which extracellular binding translates to JAK activation is limited. Studies on purified truncated proteins indicated that the IL‐23 receptor is activated through a ligand induced dimerisation mechanism, whereby IL‐23 is first bound to the IL23R followed by the recruitment of IL12Rβ1 to the IL‐23:IL23R complex (Bloch et al., 2018). However, further studies utilising recombinant cellular systems have indicated that the receptor is activated by conformational change of pre‐associated complexes (Lay et al., 2022; Sivanesan et al., 2016).

In this study, NanoLuc Binary Technology (NanoBiT) was utilised to measure the formation of IL‐23 receptor complexes in the presence and absence of the cytokine, to assess if heteromers form on the cell surface prior to ligand engagement. The NanoBiT methodology relies on a split version of the deep‐sea shrimp derived bioluminescent enzyme NanoLuciferase (NanoLuc). The enzyme is split into a large (18 kDa) fragment (LgBit) and smaller (1.3 kDa) high affinity (HiBit) or low affinity (SmBit) peptides (Dixon et al., 2016). Fusions of NanoBiT fragments to the termini of targets of interest can be used to monitor the formation and disruption of protein complexes through changes in the production of bioluminescence by the complemented enzyme (Dale et al., 2019).

## 2 METHODS

### 2.1 Materials

Tetramethylrhodamine (TMR) tagged IL‐23 (IL23‐TMR) was generated as described in Lay et al. (2022). Recombinant IL‐23 was obtained from GlaxoSmithKline (Stevenage, UK). Fugene HD, HaloTag Alexa Fluor 488 and the Nano‐Glo luciferase assay system were purchased from Promega (Madison, USA). Fetal bovine serum, protease‐free bovine serum albumen, Dulbecco's modified Eagle's medium, poly‐d‐lysine hydrobromide and phosphate‐buffered saline (PBS) were obtained from Sigma‐Aldrich (Gillingham, UK). Opti‐MEM reduced serum medium was purchased from Thermo Fisher Scientific (Loughborough, UK). The Alpha‐Lisa Sure Fire Ultra p‐STAT3 (Tyr705) assay kit was obtained from Perkin Elmer (Waltham, USA). HEK293T cells (female; RRID:CVCL_0063) were purchased from ATCC (Virginia, USA). pcDNA3.1 zeo was purchased from Thermo FIsher Scientific and the IL23R‐MycDDK, and IL12Rβ1 expression plasmids were obtained from Origene (Rockville, USA). The pcDNA3.1 vectors containing *N‐*terminal HiBit, SmBit and LgBit were generated as described in Soave, Heukers, et al. (2020) and Soave, Kellam, et al. (2020). The IL6SS‐NL‐IL23R, IL6SS‐HT‐TEV‐IL12Rβ1 and IL6SS‐HT‐TEV‐IL23R expression constructs were purchased from GenScript (Piscataway, USA) as described in (Lay et al., 2022).

### 2.2 Creation of NanoBiT constructs

HiBit‐IL23R and SmBit‐IL23R expression constructs were generated by restriction cloning IL23R into pc.3.1 expression vectors containing *N‐*terminal HiBit or SmBit (kindly donated by Dr Mark Soave; Soave, Kellam, et al., 2020), using restriction enzymes Xba1 and BamH1. This resulted in linkers with the sequence SSGGSSGGSTSPVWWNSADIQHSGGRSR. LgBit‐IL23R and LgBit‐IL12Rβ1 expression plasmids were generated in a similar manner by restriction cloning IL23R and IL12Rβ1 into a pc3.1 expression vector containing *N‐*terminal LgBit with a murine 5‐HT3a secretion signal (kindly donated by Dr Mark Soave; Soave, Kellam, et al., 2020), again using Xba1 and BamH1. This procedure left a linker with the sequence GSSGEDLYFQSGSTSPVWWNSADIQHSGGRSR. HiBit‐IL23R with a truncated linker was created by site directed deletion of an 18‐amino acid portion of the linker from the previously generated HiBit‐IL23R plasmid using a site‐directed mutagenesis kit (Thermo Fisher Scientific) and phosphorylated primers specific to the regions adjacent to the deletion. The resulting construct had a linker with the sequence SSGGSSGGST. The generation of *N*‐terminal LgBit VEGFR‐2 has been described previously in Peach et al. (2021).

### 2.3 Cell culture

Human embryonic kidney (HEK) 293T cells were cultured in Dulbecco's modified eagle medium (DMEM) with 10% fetal bovine serum (FBS) at 37.5°C with 5% CO_2_. All experimental incubations were carried out in these conditions unless Hank's buffered saline solution (HBSS) was used in which case incubations were undertaken at 37.5°C without CO_2_.

### 2.4 Transient transfections

HEK293T cells were either transiently transfected in 6‐ or 96‐well microplates. For 6‐well batch transfections, cells were seeded at 200,000 cells ml^−1^ in 2 ml of media and incubated for 4–6 h. A transfection mix was made up containing a 1:4 ratio of IL12Rβ1 to IL23R constructs with a total DNA concentration of 2 μg per well (made up to the final mass with pc3.1 zeocin empty vector if only one construct was to be expressed) and 6 μl per well of FuGENE HD transfection reagent, in OptiMEM media without phenol red (total volume 100 μl per well). Six‐well plates were incubated overnight before cells were suspended, adjusted to 300,000 cells ml^−1^ and dispensed into poly‐d‐lysine (PDL) coated white clear bottom 96‐well microplates (Greiner Bio‐One, Stonehouse, UK) at 100 μl per well, and experiments were then performed the following day. For LV200 imaging experiments, cells were seeded at 150,000/ml into PDL coated 35‐mm glass bottom plates and transfected as described. These cells remained in the dish and were imaged after incubation for two further nights.

In the case of 96‐well microplate transfections, cells were suspended and adjusted to 200,000 cells ml^−1^ and dispensed into PDL coated white clear bottom microplates at 100 μl per well. The cells were then incubated overnight before being transfected the following day with a 1:4 ratio of IL12Rβ1 to IL23R constructs using 40 ng of each IL23R construct (80 ng if two IL23R constructs were co‐transfected), normalised to a total of 100 ng per well with pc3.1 zeocin empty vector, with 0.6‐μl FuGENE HD transfection reagent and made up to 5 μl per well in OptiMEM without phenol red. For AlphaLISA experiments, 80 ng of each IL23R construct and 20 ng of each IL12Rβ1 were used. These cells were then incubated overnight before experiments were carried out for following day.

Six‐well batch transfections were employed for experiments where cells were treated with a concentration titration of protein. These included NanoBRET ligand binding and competition experiments, LgBit and HiBit binding curves and AlphaLISA IL‐23 dose–response experiments. All other experiments utilised the 96‐well transfection methodology.

### 2.5 Measurement of luminescence from NanoBiT complemented cells

Ninety‐six‐well microplates containing transfected cells had media removed and replaced with HBSS with 1 mg·ml^−1^ bovine serum albumin (BSA) with or without purified IL‐23, HiBit or LgBit protein. Cells were then incubated for 1 h, before 5 μl of 77‐μM furimazine was added to each well. The plate was incubated for 5 min before a white plate back was added, and luminescence readings were measured using a PheraStar FS plate reader (BMG Labtech) with gains of 3600.

### 2.6 Comparison of HiBit‐IL‐23R complementation with either IL12Rβ1 or vascular endothelial growth factor receptor 2 (VEGFR‐2)

HEK293T wildtype cells were seeded at 20,000 cells per well into PDL coated white clear bottom microplates at 100 μl per well. Cells were left to grow for 24 h at 37°C/5% CO_2_ to reach 50–70% cell confluency. Cells were then transfected with the appropriate cDNA constructs using FuGENE HD at a 3:1 reagent:DNA ratio in OptiMEM following manufacturer's instructions. All cDNA constructs were initially diluted to a concentration of 50 ng·μl^−1^ in OptiMEM. HiBiT tagged IL‐23R was used at a final concentration of 80 ng per well, with LargeBiT (LgBit) tagged receptor partners (IL‐12Rβ1 or VEGFR‐2) co‐transfected at a concentration of 20 ng per well. All wells were transfected with a total DNA concentration of 100 ng per well, with empty pcDNA 3.1 vector used to ensure consistency in total DNA concentration between wells. For each transfection, triplicate wells were prepared for each experiment. Following transfection, plates were incubated at 37°C/5% CO_2_ for a further 18 h. On the day of assay, well contents were replaced with HBSS/0.1% BSA (warmed to 37°C) in the presence or absence of 50‐nM purified HiBit. Cells were then incubated at 37°C for 20 min, followed by the addition of a 1:400 dilution (final well concentration) of furimazine to all wells. Plates were then left in the dark for 5 min before total luminescence detected using a BMG Pherastar with gains of 3600.

### 2.7 Luminescence imaging

Media was removed from dishes containing transiently transfected cells and replaced with HBSS containing 5.13 μM of furimazine (5.78 μM of furimazine was used in NanoBiT experiments). Brightfield and luminescence images were then taken of the dishes using an LV200 luminescence microscope (Olympus) equipped with a C9100‐23B IMAGE EMX2 camera (Hamamatsu) and a 60×/1.42NA oil immersion objective with 0.5× tube lens.

### 2.8 NanoBiT BRET experiments

Transfected cells in 96‐well microplates had media removed and replaced with a concentration titration of IL23‐TMR in HBSS with 1 mg·ml^−1^ BSA with or without IL‐23. The cells were incubated for an hour before 5 μl of 77‐μM diluted furimazine was added to each well. The plate was then incubated for 5 min, a white plate back was added and the BRET signal was measured on a PheraStar FS plate reader using a 450‐nm (30‐nm bandpass) and >550‐nm filter with gains of 2800 and 3600, respectively.

### 2.9 Measurement of STAT3 phosphorylation

AlphaLISA Surefire Ultra assays were carried out as previously described (Lay et al., 2022). Briefly, media was replaced with 100‐μl DMEM without FBS, and the cells incubated for 3 h. Media was then replaced with 50‐μl HBSS with 1 mg·ml^−1^ BSA, with or without IL‐23 and cells incubated for a further 30 min. The assay was then carried out according to the manufacturer's instructions.

### 2.10 Measurement of HaloTag fluorescence intensity

Cells were incubated with media containing 200‐nM Alexa Fluor (AF) 488 HaloTag ligand for 30 min. Cells were then washed three times with 50‐μl HBSS followed by the addition of 50‐μl HBSS with 1 mg·ml^−1^ BSA and measurement of fluorescence intensity using a PheraStar FS plate reader using an excitation laser at 485 nm and emission at 520 nm.

### 2.11 Data analysis

Data are presented as mean ± SEM. All experiments were performed in at least five independent experiments with triplicate or quadruplicate wells (see figure legends for details). Drug additions were randomly allocated to wells within a 96‐well plate.

Scale bars were added to luminescence images using FIJI (National Institute of Health). BRET ratio values were generated in MARS (BMG Labtech) using the below equation:

$$\text{BRET ratio}=\frac{\text{Acceptor signal}}{\text{Donor signal}\;}.$$

Data were exported from the PheraStar FS reader and stored in Microsoft Excel. Further data analysis was carried out using GraphPad Prism.

BRET Ligand binding curves were fitted with the GraphPad Prism ‘One site – Total and nonspecific binding’ fit, which used the equation:

$$\text{BRET ratio}=\frac{B_{\max}\left[A\right]}{\left(\left[A\right]+K_{D}\right)}+\left(\left(B\left[A\right]\right)+C\right),$$

where ‘B_max_’ is the maximum BRET signal, ‘[A]’ is IL23‐TMR concentration, ‘B’ is the non‐specific binding component's slope and ‘C’ is the Y intercept.

Competition curves were fitted with the GraphPad Prism ‘log (inhibitor/agonist) vs. response – Variable slope (four parameters)’ fit which used the equation:

$$Y=B_{\min}+\frac{B_{\max}-B_{\min}}{1+10^{\left(\text{LogXC}50-\left[A\right]\right)C}},$$

where ‘B_max_’ is the maximum signal, ‘B_min_’ is the minimum signal, ‘LogXC50’ is the log of the 50% inhibiting concentration for competition experiments and 50% agonising concentration in STAT3 phosphorylation experiments, ‘[A]’ is the concentration of IL‐23 and ‘C’ is the Hill slope.

Normalised binding curves for HiBit and LgBit were fit using the GraphPad Prism ‘One site – Specific binding’ fit using the equation:

$$Y=\frac{B_{\max}\left[B\right]}{\left(\left[B\right]+K_{D}\right)},$$

where ‘B_max_’ is the maximum signal of the curve and ‘[B]’ is the concentration of IL23‐TMR.

The data and statistical analysis comply with the recommendations of the British Journal of Pharmacology on experimental design and analysis in pharmacology (Curtis et al., 2022). Statistical analysis was undertaken only for studies where each group size was at least n = 5. A value of *P* < 0.05 was used to determine statistical significance.

### 2.12 Nomenclature of targets and ligands

Key protein targets and ligands in this article are hyperlinked to corresponding entries in http://www.guidetopharmacology.org, and are permanently archived in the Concise Guide to PHARMACOLOGY 2021/22 (Alexander, Fabbro et al., 2021a, 2021b; Alexander, Kelly et al., 2021a).

## 3 RESULTS

### 3.1 Use of NanoBiT to monitor IL23R‐IL12Rβ1 heteromer formation and ligand‐binding

**FIGURE 1:**
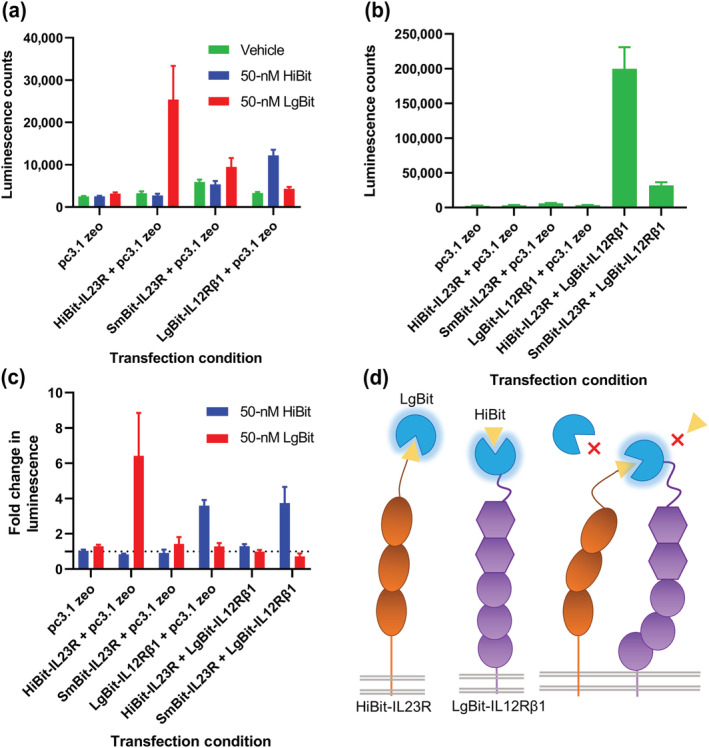
NanoBiT tagged IL‐23 receptor complexes associate on the cell surface in the absence of ligand. (a) The luminescence generated when HEK293T cells transiently transfected with NanoBiT conjugated IL‐23 receptor constructs was incubated with purified NanoBiT reagents. (b) The luminescence signal observed when complementarily tagged NanoBiT constructs was co‐expressed together. (c) The fold change in luminescence from the signal observed in (b) when cells was incubated with purified HiBit or LgBit protein. (d) A schematic demonstrating the results of (c). Data are mean ± SEM from five independent experiments, which each contained quadruplicate determinations.

Plasmid DNA constructs containing *N‐*terminal HiBit, SmBit and LgBit fusions of IL23R and HiBit and LgBit fusions of IL12Rβ1 were created. These constructs were then transiently expressed in HEK293T cells to test that they were surface expressed and that they retained the ability to be complemented by corresponding NanoBiT fragments. Figure 1a demonstrates that each of these complexes generated little luminescence when expressed alone and supplied with the NanoLuciferase substrate furimazine. However, when exogenous purified HiBit or LgBit protein was added, luminescence was produced in the presence of the corresponding fragment expressed on the *N*‐termini of an IL‐23 receptor monomer. The highest signal was observed for HiBit‐IL23R complemented with LgBit, with a lower signal observed for the SmBit‐IL23R construct due to the lower affinity of SmBit for LgBit (Peach et al., 2021).

When HiBit‐IL23R was expressed with LgBit‐IL12Rβ1, a strong luminescent signal was observed (73.3‐fold that of the mean of the receptor constructs expressed in isolation). When an equivalent pairing was expressed containing SmBit‐IL23R, the signal was weaker (6.19‐fold lower) but remained higher than that of the monomers complemented with exogenous protein (10.3‐fold higher than the mean of receptor constructs expressed in isolation; Figure 1b). Finally, when cells expressing the complemented receptor pairs were exposed to purified HiBit and LgBit protein (Figure 1c,d), there was no increase in luminescence in either condition for HiBit‐IL23R containing heteromers. There was however an increase in luminescence equivalent to that observed for LgBit‐IL12Rβ1 expressed alone when purified HiBit was added to cells expressing the SmBit‐IL23R containing heteromer, indicating that this construct had a lower affinity for LgBit‐IL12Rβ1 than the equivalent HiBit construct.

**FIGURE 2:**
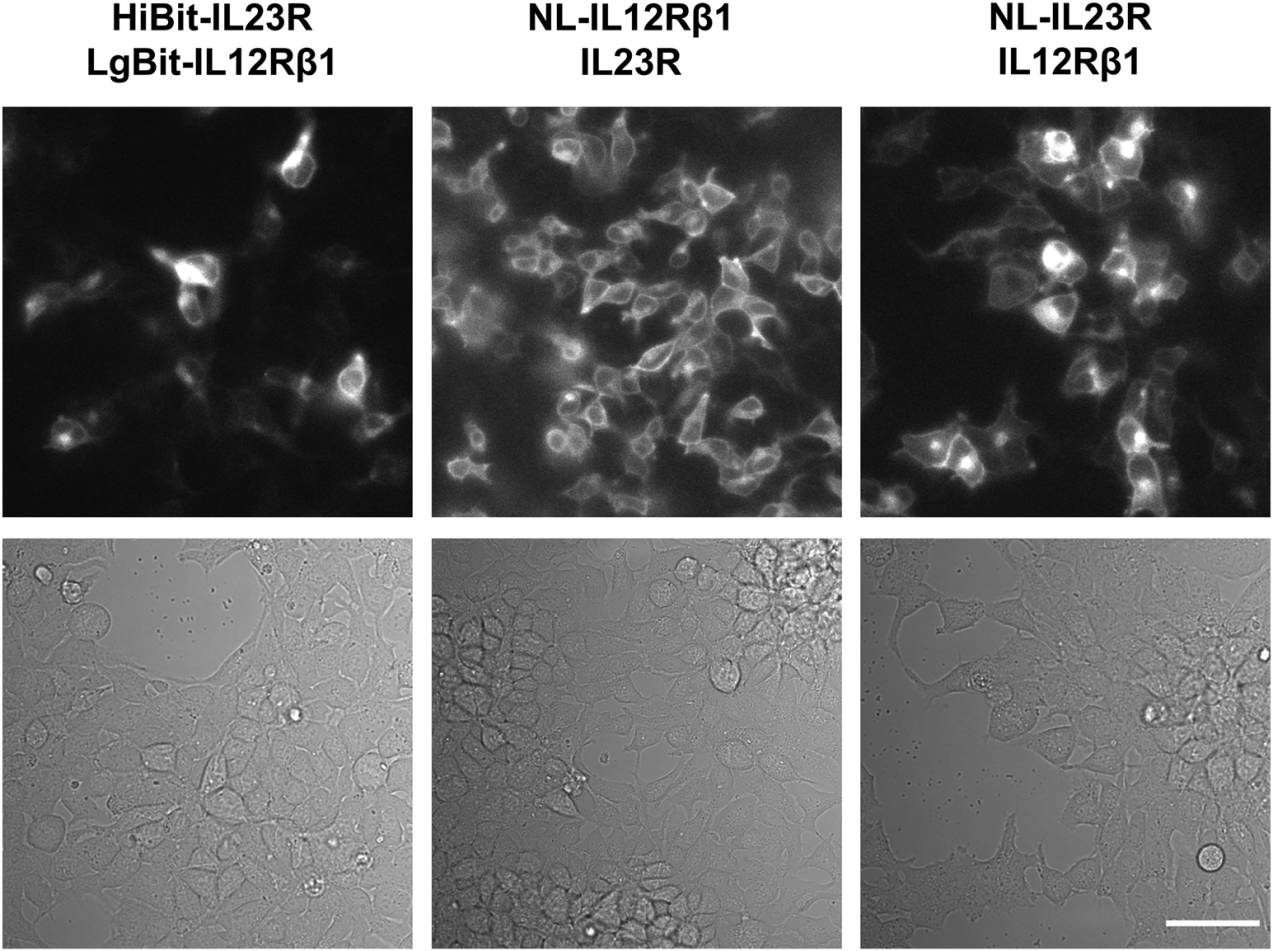
Luminescence microscopy demonstrates localisation of NanoBiT heteromers. Luminescence (top) and brightfield (bottom) images of HEK293T cells transiently expressing IL‐23 receptor (IL23R) fusions. Images are representative of four (HiBit‐IL23R + LgBit‐IL12Rβ1) or five independent experiments. Scale bar represents 50 μm.

To assess the localisation of NanoBiT complemented heteromers, luminescence images were taken of cells expressing HiBit‐IL23R and LgBit‐IL12Rβ1. These images demonstrated luminescence at the periphery of the cell, and at intracellular localisations, these observations were similar to NanoLuc (NL)‐IL23R when imaged with IL12Rβ1 under equivalent conditions (Figure 2).

**FIGURE 3:**
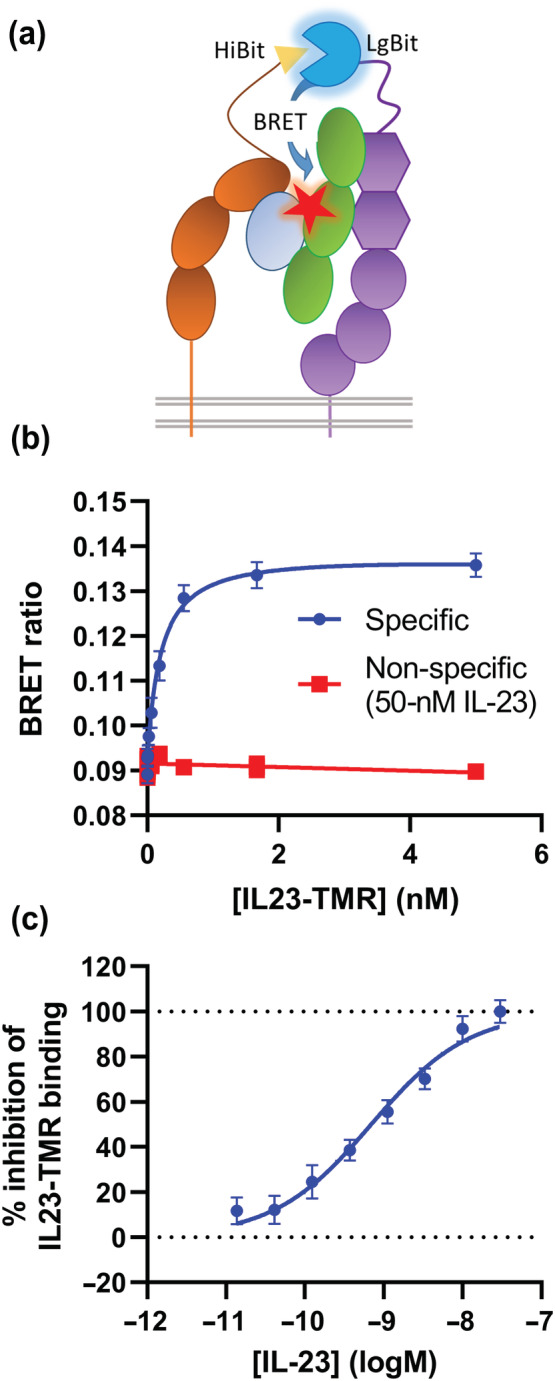
NanoBiT complemented receptor heteromers bind IL‐23 with similar affinity to NanoLuciferase tagged heteromers. (a) A schematic depicting the NanoBiT BRET assay used in (b) and (c). (b) The NanoBRET signal generated when increasing concentrations of IL23‐TMR was applied to cells expressing HiBit‐IL23R and LgBit‐IL12Rβ1. (c) The inhibition of 300‐pM IL23‐TMR binding to cells expressing HiBit‐IL23R and LgBit‐IL12Rβ1 by increasing concentrations of IL‐23. Data are mean ± SEM from five independent experiments, which each contained triplicate determinations.

**FIGURE 4:**
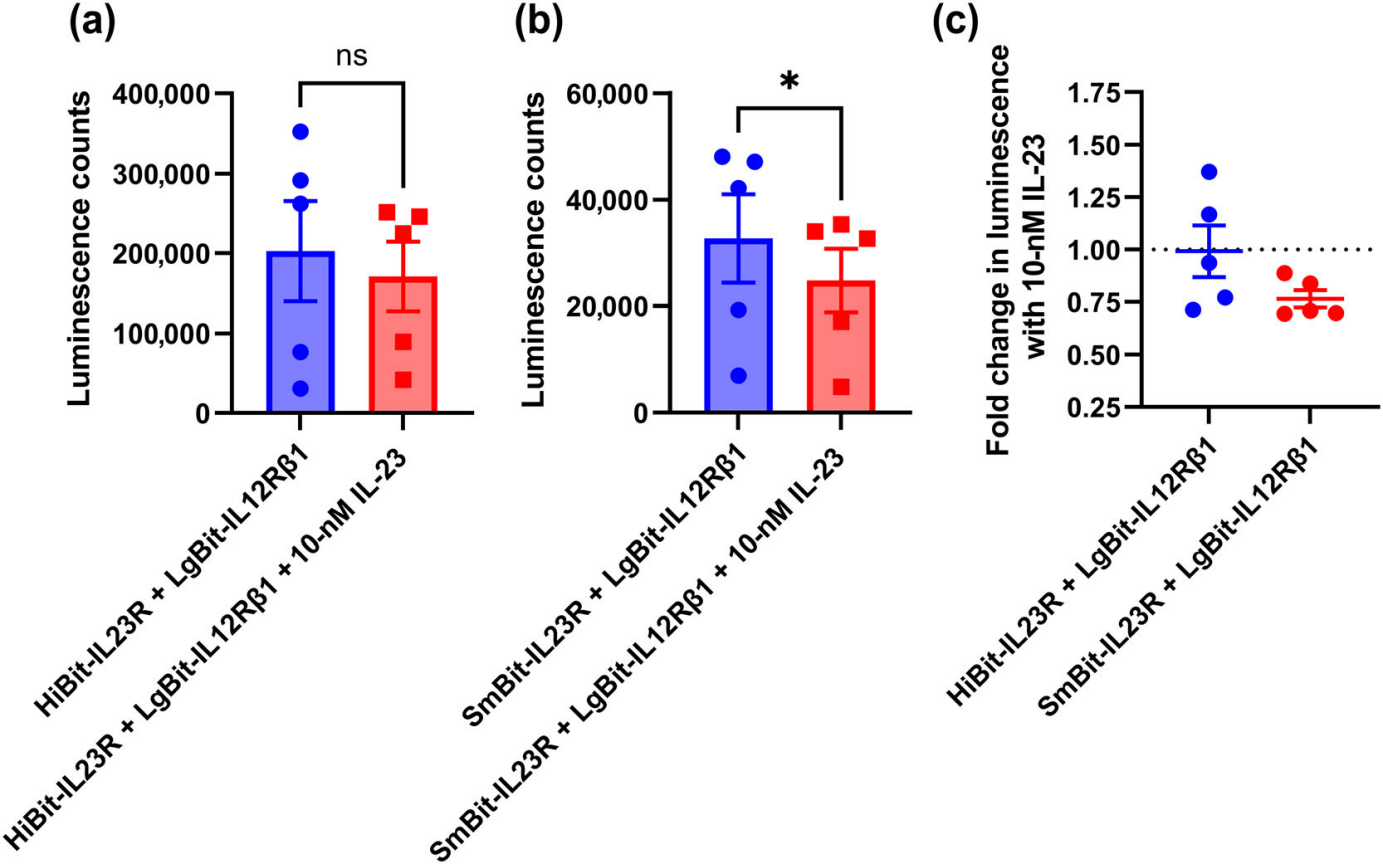
NanoBiT heteromers bind IL‐23 without any increase in complex formation. (a, b) The luminescence of cells expressing (a) HiBit‐IL23R or (b) SmBit‐IL23R and LgBit‐IL12Rβ1 with or without treatment with 10‐nM IL‐23. Statistical significance (*p*  <0.05; paired *t* test) is indicated by *. (c) The fold change in luminescence generated when cells expressing HiBit or SmBit containing NanoBiT complemented heteromers is incubated with 10‐nM IL‐23. Data are mean from five independent experiments conducted in quadruplicate with overall mean ± SEM superimposed.

Previously, we created and validated a TMR labelled IL‐23 species termed IL23‐TMR (Lay et al., 2022). To test if the NanoBiT heteromers were able to bind ligand, IL23‐TMR was applied to cells expressing HiBit or SmBit‐IL23R and LgBit‐IL12Rβ1 before measuring a NanoLuc Bioluminescence Resonance Energy Transfer (NanoBRET) signal generated between the complemented luciferase and IL23‐TMR. The luminescence signal generated from the SmBit heteromer was too low to produce a reliable BRET ratio; however, a robust BRET signal was generated using cells transfected with the HiBit heteromer, which could be abolished through the addition of unlabelled IL‐23 (Figure 3). The affinity of IL23‐TMR for these binary IL‐23 receptors was measured to be 234 ± 40 pM (Figure 3b, mean ± SEM, n = 5). This value was between that of the previously measured values for IL23‐TMR binding to HEK293T cells expressing NL‐IL23R and IL12Rβ1 (27.0 ± 3.6 pM) and cells expressing IL23R and NL‐IL12Rβ1 (647 ± 80 pM; Lay et al., 2022). To confirm that non‐modified IL‐23 could also bind to the NanoBiT complemented heteromers, we carried out a NanoBRET competition assay to measure the displacement of 300 pM IL23‐TMR from cells expressing HiBit‐IL23R and LgBit‐IL12Rβ1 by increasing concentrations of IL‐23 (Figure 3c). An IL‐23 affinity of 352 ± 155 pM (mean ± SEM, n = 5) was measured, which was comparable to that measured for IL23‐TMR. Whilst it was confirmed that both IL23‐TMR and IL‐23 bound to the NanoBiT heteromers, there was no increase in NanoBiT complementation when IL‐23 was added to either SmBit or HiBit heteromers (Figure 4). On the contrary, the addition of IL‐23 significantly (paired *t* test, *p* = <0.05) reduced the complementation of SmBit heteromers (Figure 4b), indicating that ligand binding reduced the proximity of the *N‐*terminal domains of the receptor.

### 3.2 Use of NanoBiT to monitor IL23R homomer formation and ligand‐binding

**FIGURE 5:**
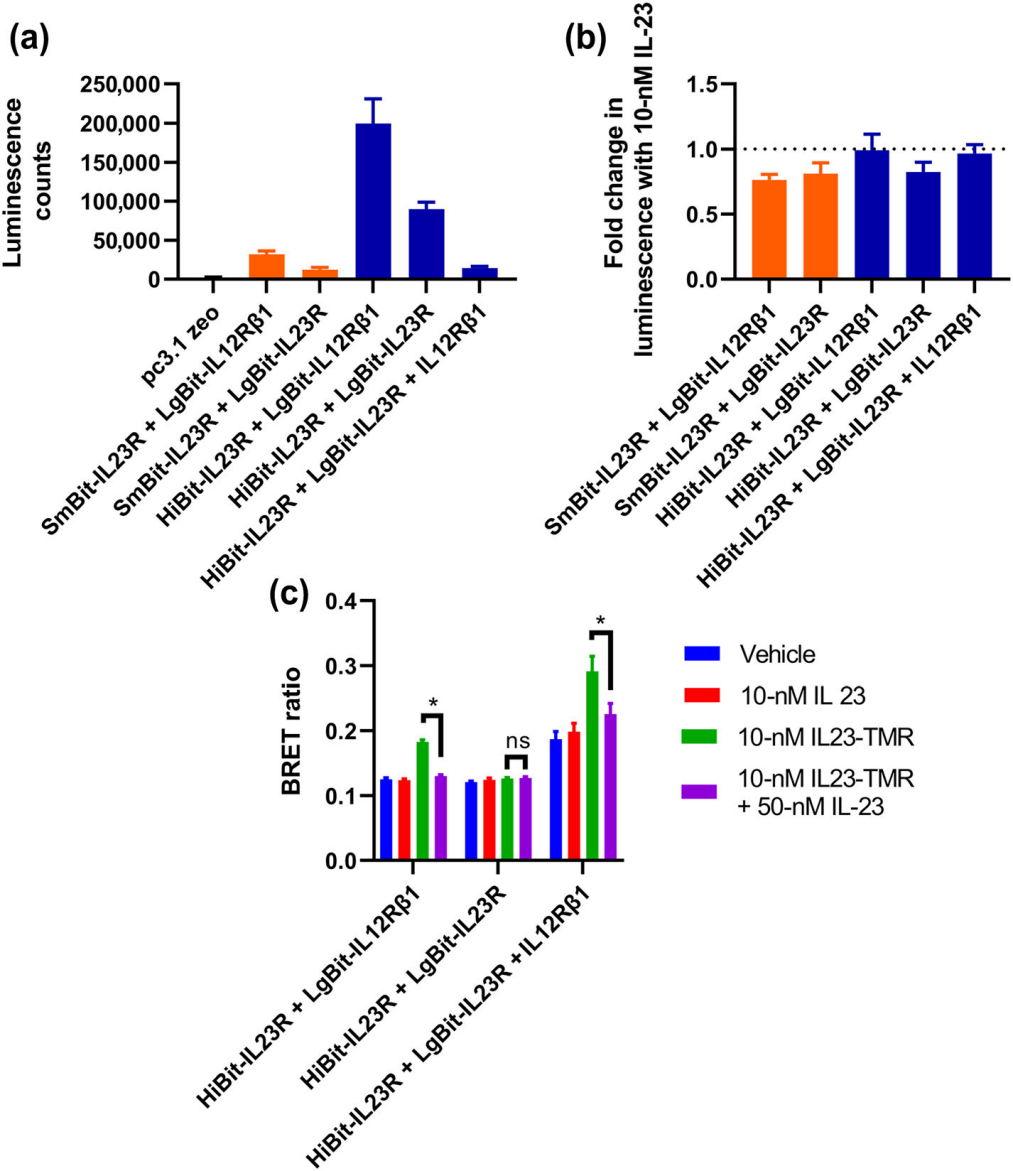
NanoBiT linked homomers of IL23R but not IL12Rβ1 can engage IL‐23 when co‐expressed with their co‐receptor. (a) The luminescence generated from cells transfected with different combinations of NanoBiT constructs. (b) The fold change in luminescence when the cells from conditions in (a) is treated with 10‐nM IL‐23. (c) The BRET ratio generated when the cells from (a) are treated with IL23‐TMR. Data are mean ± SEM from five quadruplicate independent experiments. Statistical significance (*p* <0.05; paired *t* test) is indicated by *.

The luminescent signal generated by homomeric pairings of IL23R was also tested (Figure 5a). A luminescent signal was generated by both the HiBit and SmBit homomeric pairings of IL23R indicating that complexes were forming containing at least two IL23R proteins; however, the luminescence signal was half the amplitude (HiBit 2.12‐fold and SmBit 2.18‐fold) of the equivalent heteromeric condition. The HiBit homomer signal was also stronger (6.36‐fold) than that of the SmBit homomer. Co‐expression of IL12Rβ1 with HiBit complemented IL23R homomers reduced the signal measured (6.73‐fold). Treatment of IL23R homomers with IL‐23 in the presence or absence of their co‐receptors did not lead to significant changes in luminescence (paired *t* test; Figure 5b).

**FIGURE 6:**
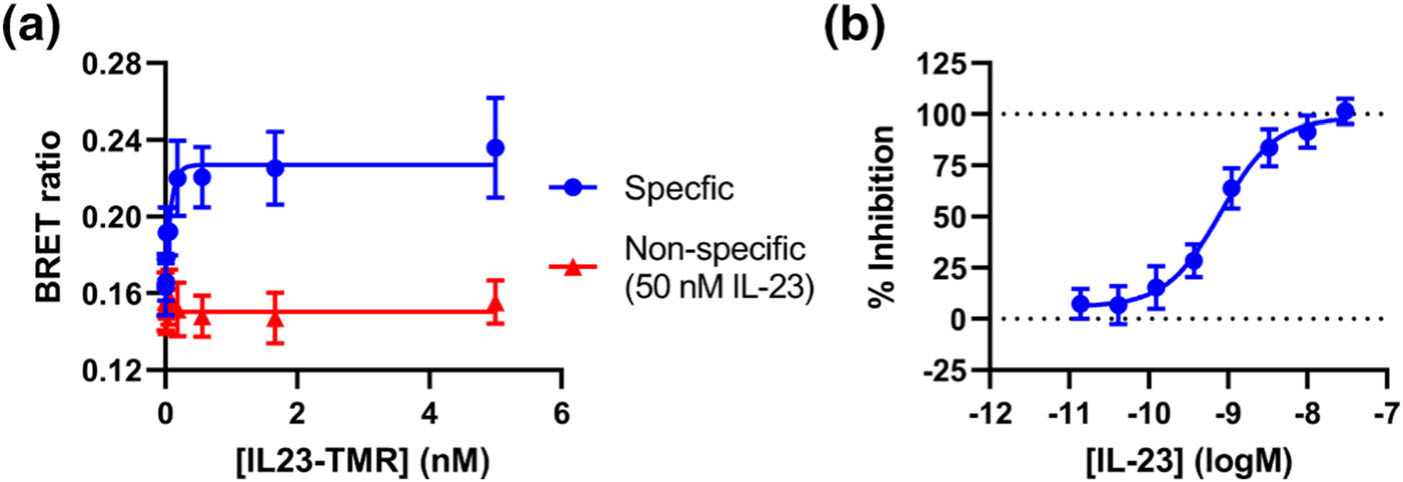
NanoBiT IL23R homomers can engage IL‐23 when co‐expressed with IL12Rβ1. (a) The BRET signal generated when cells expressing HiBit‐IL23R, LgBit‐IL23R and IL12Rβ1 was treated with a concentration titration of IL23‐TMR. (b) The inhibition of 300‐pM IL23‐TMR binding to cells expressing HiBit‐IL23R, LgBit‐IL23R and IL12Rβ1 by a concentration titration of IL‐23. Data are mean ± SEM from five independent experiments, each performed in triplicate.

Homomers of HiBit‐IL23R and LgBit‐IL23R did not generate a BRET signal when treated with 10 nM IL23‐TMR (Figure 5c); however, when cells were co‐transfected with HiBit‐IL23R, LgBit‐IL23R and IL12Rβ1, a BRET signal was generated that could be displaced by IL‐23 (Figure 5c). These trimeric or multimeric species had an affinity of 36.2 ± 16.5 pM for IL23‐TMR and 140 ± 67 pM for IL‐23 (Figure 6).

### 3.3 Low complementation between HiBit‐IL‐23R and LgBit‐VEGFR‐2

**FIGURE 7:**
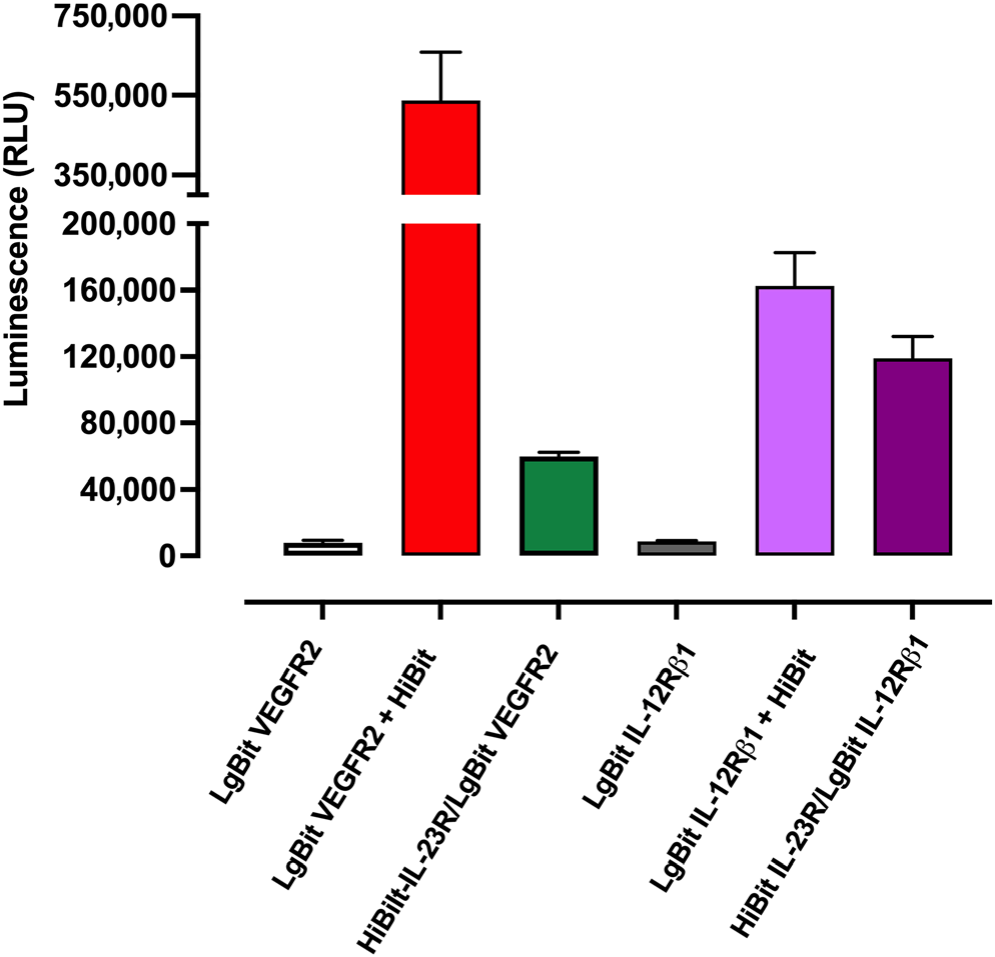
Low complementation between HiBit‐IL‐23R and LgBit‐VEGFR‐2. Luminescence generated when HEK293T cells transiently transfected with LgBit‐conjugated IL12Rβ1 or VEGFR‐2 constructs was incubated with either purified HiBit (50 nM) or co‐transfected with HiBit‐IL‐23R. The luminescence generated by LgBit‐VEGFR‐2 and LgBiT‐IL12Rβ1 in the absence of purified HiBit is also shown. Data are mean ± SEM from four independent experiments, each performed in triplicate.

To check whether complementation could occur between HiBit‐IL23R and an unrelated LgBit‐tagged receptor expressed on the surface of HEK293T cells, we investigated the association between the IL23R and LgBit‐tagged VEGFR‐2, which we have previously show to form heterodimers with its co‐receptor neuropilin‐1 (Peach et al., 2021). As we have previously described (Peach et al., 2021), LgBit‐VEGFR‐2 was well expressed on the surface of HEK293T cells following transient transfection and gave a strong luminescence signal when complemented with 50‐nM purified HiBit (Figure 7). However, when HiBit‐IL23R was co‐expressed with LgBit‐VEGFR‐2, the signal was only 11.6% of the luminescence signal obtained with LgBit‐VEGFR‐2 in the presence of 50‐nM purified HiBit (Figure 7). In parallel transfections in the same experiments, HiBit‐IL23R complementation to LgBit‐IL12Rβ1 yielded a luminescence signal that was 73% of the signal obtained with LgBit‐IL12Rβ1 in the presence of 50‐nM purified HiBit (Figure 7). These data suggest that a small HiBit‐IL23R complementation can occur when unrelated LgBit‐tagged cell surface receptors are expressed at a high level. However, in the case of interactions between HiBit‐IL23R and LgBit‐IL12Rβ1, a large percentage of the LgBit‐IL12Rβ1 receptors expressed at the cell surface are complemented to HiBit‐IL23R.

### 3.4 Ligand independent STAT3 phosphorylation can be induced with specific orientations of high affinity NanoBiT complementation

**FIGURE 8:**
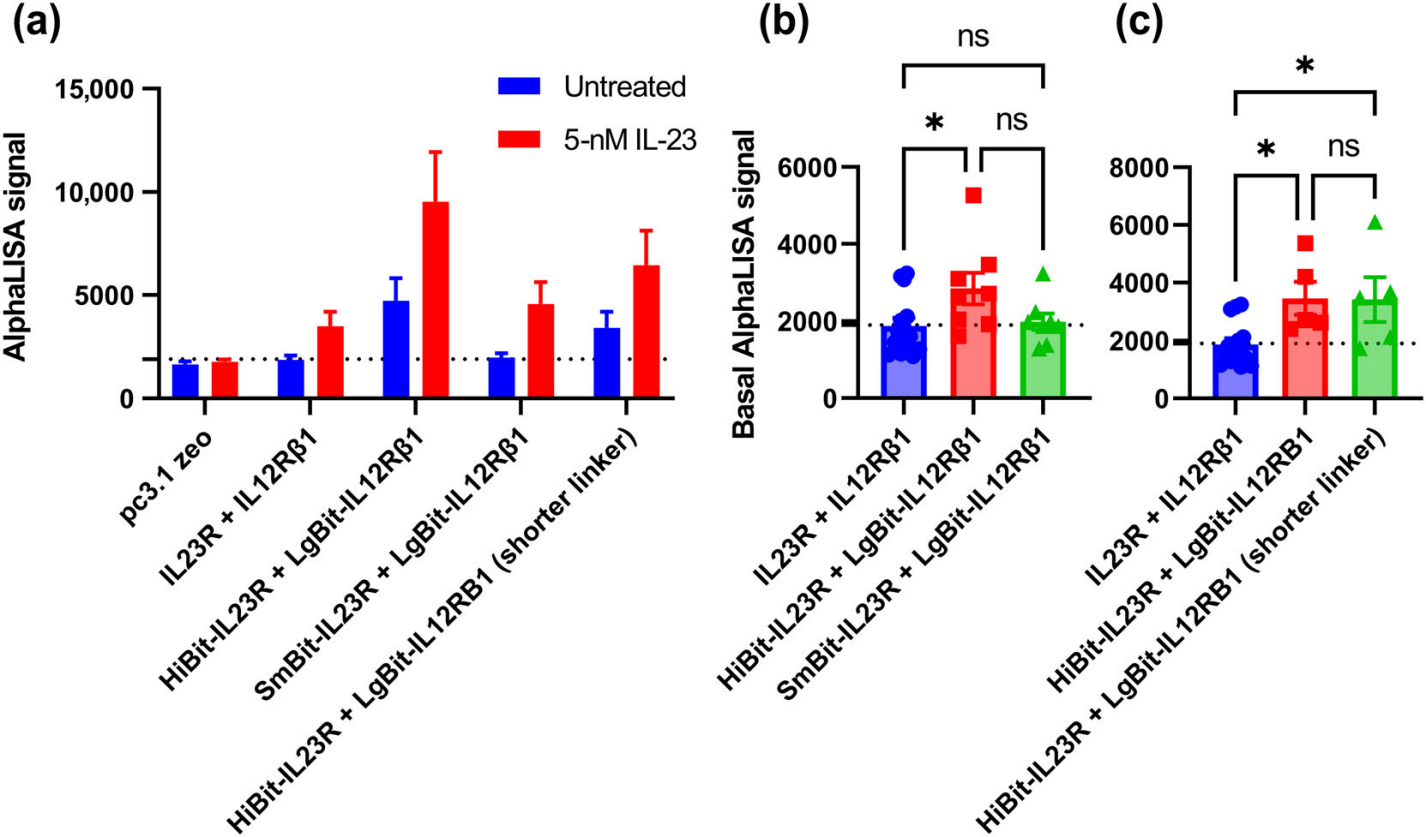
High affinity NanoBiT complementation induces ligand independent signalling. (a) The phospho‐STAT3 AlphaLISA signal generated from cells transfected with NanoBiT constructs and treated with IL‐23 for 30 min. Data are mean ± SEM from 5 (HiBit‐IL23R with a short linker), 8, 14 (wildtype) or 17 (HiBit‐IL23R) triplicate experiments. (b, c) Comparison of the basal pSTAT3 signalling of cells expressing HiBit‐IL23R and LgBit‐IL12Rβ1 with either (b) cells expressing SmBit‐IL23R and LgBit‐IL12Rβ1 or (c) cells expressing LgBit‐IL12Rβ1 and a HiBit‐IL23R with an 18 amino acid truncated linker. Data are mean values generated in eight (b) or five (c) independent matched experiments with previously generated wildtype data for comparison; overall mean and SEM are also plotted. A one‐way ANOVA was used to measure the statistical significance of differences. * signifies *p* <0.05.

An AlphaLISA assay for STAT3 phosphorylation was used to test the effect of NanoBiT complementation on signal transduction. The results demonstrated that both HiBit and SmBit heteromers induced STAT3 phosphorylation when cells were treated with IL‐23 (Figure 8a). When increasing concentrations of IL‐23 were applied to cells expressing the HiBit‐IL23R and LgBit‐IL12Rβ1, the EC_50_ of STAT3 was measured to be 193 ± 69 pM (n = 4; data not shown), which was similar to the previously measured value for cells transfected with wildtype receptor in an equivalent assay (269 ± 106 pM; Lay et al., 2022).

It was observed that, in the absence of ligand, cells transfected with HiBit‐IL23R but not SmBit‐IL23R containing heteromers had significantly higher (one‐way ANOVA, *p* <0.05) basal levels of phospho‐STAT3 than cells expressing wildtype receptor (Figure 8b). To test if this effect was directly related to the proximity of the *N*‐terminal domains of the receptor, we tested the basal STAT3 phosphorylation of cells transfected with an equivalent NanoBiT heteromer in which the linker between HiBit and IL23R had been shortened by 18 amino acids (from 28 to 10). In this tagging conformation, the basal transduction remained significantly higher than that of wildtype receptor transfected cells (Figure 8c). It was observed that cells transfected with the HiBit‐IL23R containing heteromers also had a higher IL‐23 induced amplitude of signalling than wildtype transfected cells (Figure 8a).

### 3.5 Impact of heteromerisation on the binding affinity of purified LgBit for HiBit‐IL23R

**FIGURE 9:**
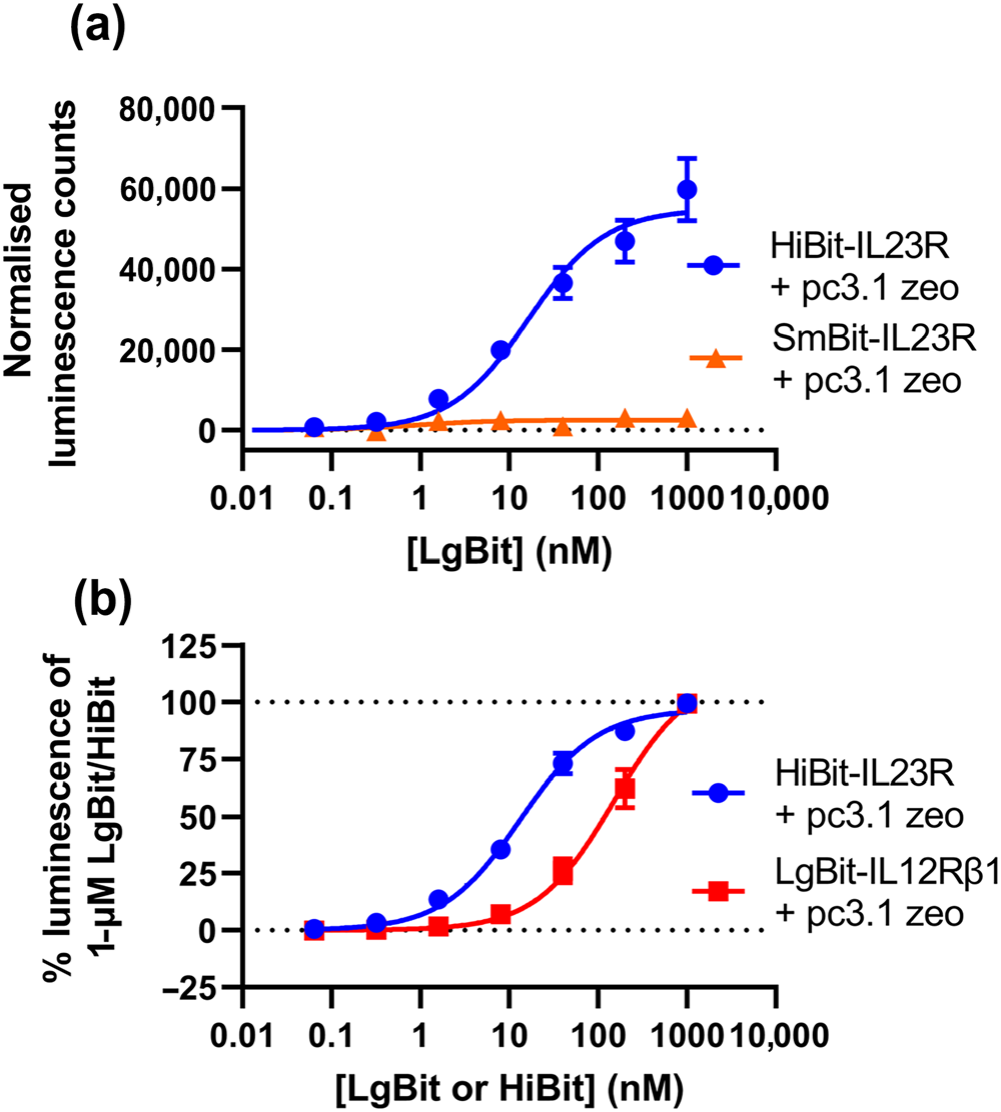
HiBit‐IL23R has a greater affinity for purified LgBit protein than SmBit‐IL23R. (a) The luminescence signal generated when a concentration titration of LgBit was applied to cells expressing either HiBit or SmBit conjugated IL23R. (b) The luminescence signal generated when increasing concentrations of purified complementary NanoBiT fragment was applied to cells transiently expressing HiBit‐IL23R or LgBit‐IL12Rβ1 in isolation. Data are mean with SEM from five or six (SmBit‐IL23R) independent experiments, each performed in triplicate.

To assess whether receptor heteromerisation alters the conformation of the *N*‐terminal regions of the partner receptor proteins, we measured whether this changed the affinity of *N‐*terminal NanoBiT‐tagged receptors for purified NanoBiT complementation partners. We first investigated the effect of applying increasing concentrations of purified HiBit or LgBit to cells expressing the HiBit‐IL23R, SmBit‐IL23R or LgBit‐IL12Rβ1, respectively (Figure 9). The HiBit‐IL23R had an affinity of 15.2 ± 1.6 nM (n = 5) for purified LgBit, which was 21.7‐fold weaker than the value quoted for HiBit alone (Dixon et al., 2016). The affinity of purified LgBit for cells expressing the SmBit‐IL23R was also tested; however, no luminescence signal was detected over the concentration range used; this observation is in accordance with the reported 190‐μM affinity of SmBit for LgBit (Dixon et al., 2016). When the affinity of purified HiBit control protein for LgBit‐IL12Rβ1 was measured it was found that whilst there was an increase in luminescence, this signal did not saturate over the concentration range tested (up to 1 μM) (Figure 9b).

**FIGURE 10:**
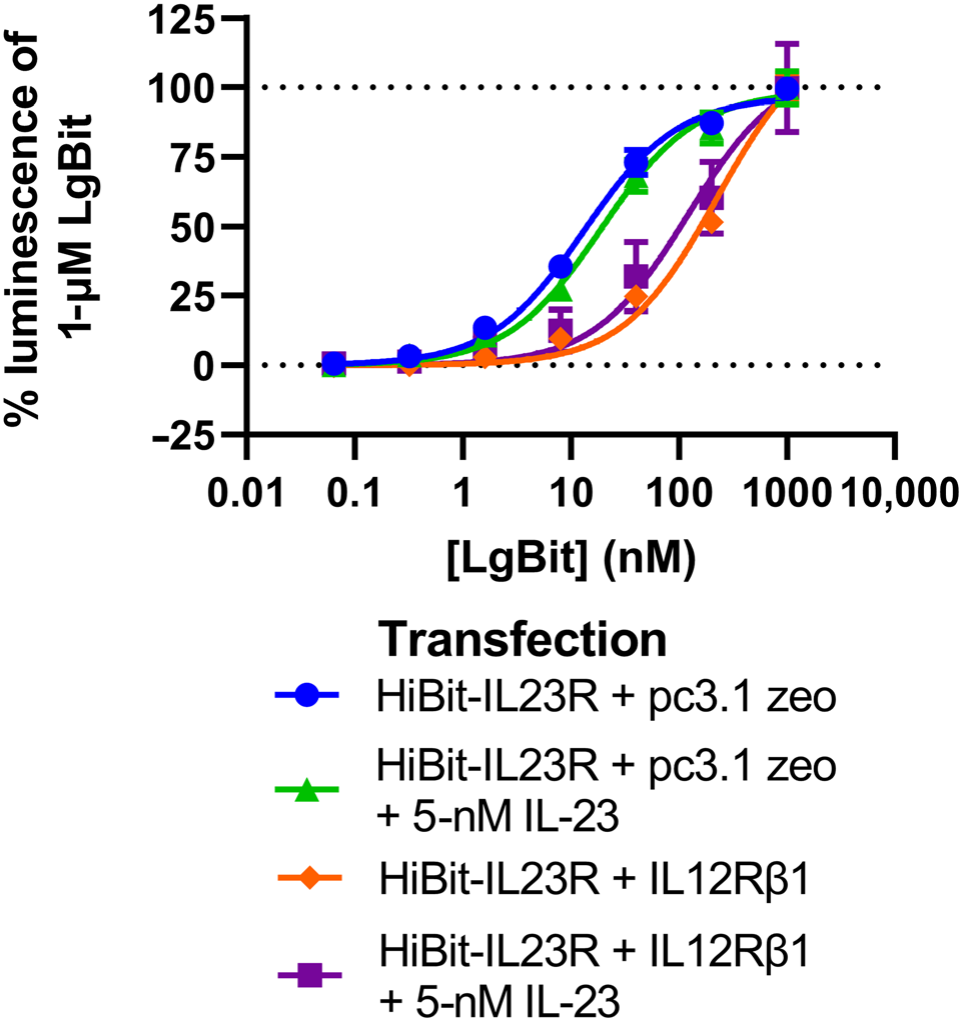
Heteromerisation but not ligand engagement decreases the affinity of exogenous LgBit for HiBit‐IL23R. The luminescence signal generated by the application of increasing concentrations of purified LgBit protein to cells expressing HiBit‐IL23R in the presence or absence of co‐expressed IL12Rβ1 and with or without IL‐23. Data are mean ± SEM from five independent experiments, each performed in triplicate.

To probe if the affinity of the receptor conjugated NanoBiT tags was influenced by heteromerisation, assays were carried out to measure the affinity of HiBit‐IL23R for purified LgBit protein when expressed in isolation or co‐expressed with untagged IL12Rβ1 and in the presence and absence of IL‐23 (Figure 10). The affinity of LgBit for HiBit‐IL23R expressed in isolation, with addition of 5‐nM IL‐23 was 25.9 ± 7.9 nM (n = 5), which was not statistically different from the HiBit dissociation constant measured in the absence of IL‐23 (unpaired *t* test). In contrast, when HiBit‐IL23R was co‐expressed with IL12Rβ1, the affinity of LgBit was decreased, to a similar degree both in the presence and absence of IL‐23, to a level where there was no clear saturation in luminescence signal at the highest concentration used in the assay.

### 3.6 Competitive disruption of NanoBiT heteromers

**FIGURE 11:**
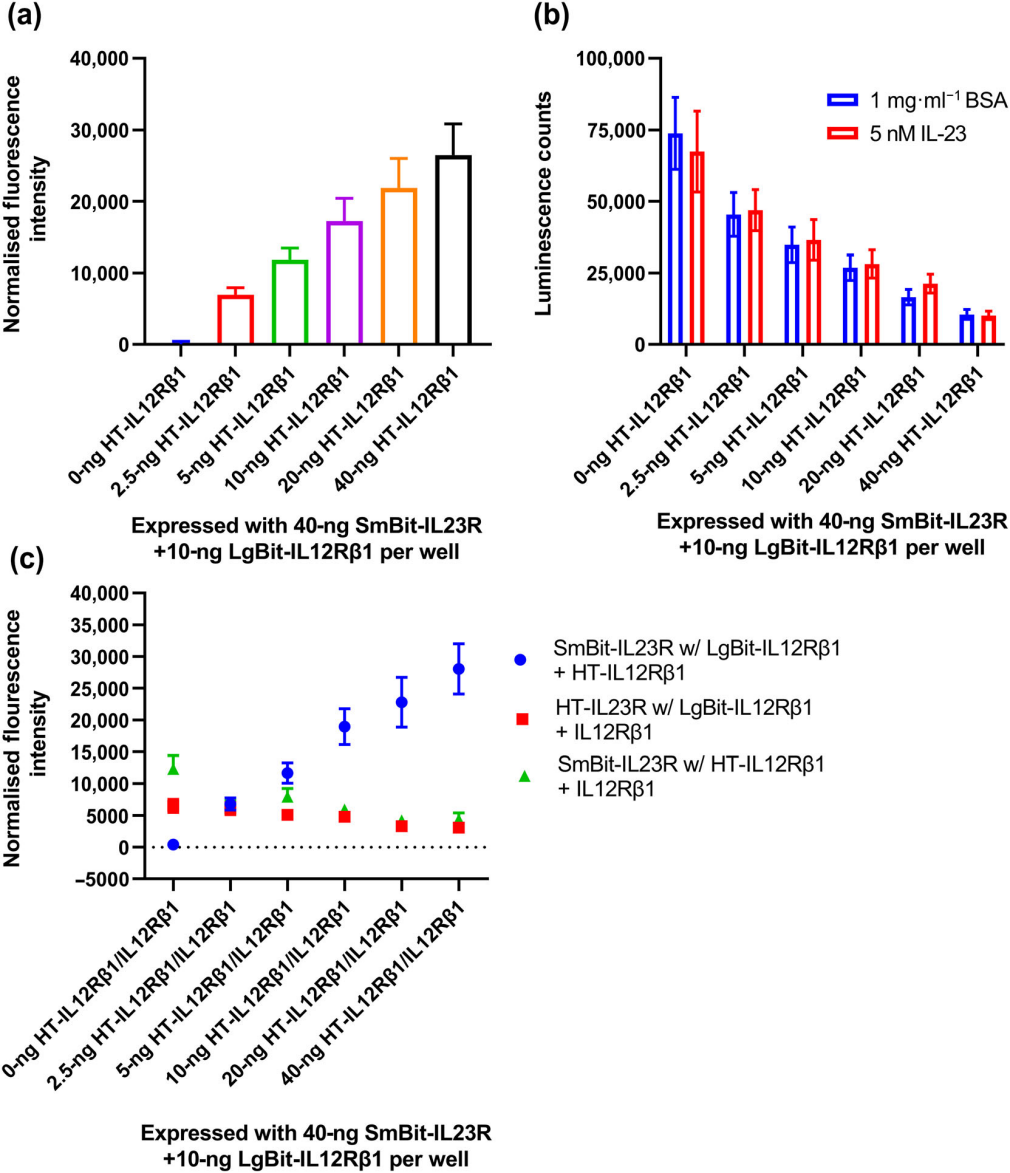
HiBit‐IL12Rβ1 competes with LgBit‐IL12Rβ1 for SmBit‐IL23R in a ligand independent manner. (a) The fluorescence intensity of cells expressing 40‐ng SmBit‐IL23R with 10‐ng LgBit‐IL12Rβ1 and increasing amounts of HT‐IL12Rβ1 cDNA, which have been labelled with HT‐AF488 ligand. (b) The luminescence signal from cells transfected as in (a) (without HT‐AF488 labelling) in the presence and absence of IL‐23. Data are mean ± SEM from five quadruplicate experiments. (c) The fluorescence intensity generated from cells labelled with HT‐AF488 and expressing 40‐ng SmBit‐IL23R cDNA, 10‐ng LgBit‐IL12Rβ1 cDNA and increasing concentrations of HT‐IL12Rβ1 (blue circles), or cells expressing increasing concentrations of IL12Rβ1 and HT‐IL23R with LgBit‐IL12Rβ1 (red squares) or SmBit‐IL23R with HT‐IL12Rβ1 (green triangles), normalised by the subtraction of the fluorescence intensity of cells expressing 50‐ng pc3.1 zeo, 10‐ng LgBit‐IL12Rβ1 and 40‐ng SmBit‐IL23R.

To test if NanoBiT heteromers of SmBit‐IL23R and LgBit‐IL12Rβ1 could be disrupted by increasing expression levels of unlabelled IL12Rβ1, cells were transfected with 40 ng of SmBit‐IL23R, 10 ng of LgBit‐IL12Rβ1 and increasing concentrations of HaloTag (HT)‐IL12Rβ1 cDNA, with the total DNA concentration normalised to 100 ng with pc3.1 zeo plasmid. Two parallel experiments were carried out; in the first condition, luminescence measurements were made in the presence and absence of IL‐23; in the second, the cells were fluorescently labelled by incubation with HT‐AF488 substrate, followed by the measurement of fluorescence intensity. These measurements showed that increasing concentrations of HT‐IL12Rβ1 cDNA led to a linear increase in HT‐IL12Rβ1 expression (Figure 11a) and that this increased expression correlated with a decrease in NanoBiT complementation (Figure 11b). Control experiments were carried out to monitor the effect of increasing the expression IL12Rβ1 on the expression of 10 ng of HT‐IL12Rβ1 or 40 ng HT‐IL23R cDNA (Figure 11c). These experiments showed that, whilst there was some decrease in expression especially in HT‐IL12Rβ1 when IL12Rβ1 expression was increased from 0 to 2.5 ng per well, the expression of receptor monomers remained relatively stable over the concentration range tested.

## 4 DISCUSSION

It has previously been suggested that inactive complexes of the IL‐23 receptor may associate in the absence of ligand (Lay et al., 2022; Sivanesan et al., 2016). In this study, heteromer formation in the absence of ligand was observed using the luminescence signal generated when *N*‐terminal domains of the receptor are labelled with corresponding NanoBiT fragments and co‐expressed in HEK293T cells. The lack of subsequent STAT3 phosphorylation observed for SmBit‐IL23R:LgBit‐L12Rβ1 heteromeric complexes confirms that heteromerisation alone is not sufficient to induce signalling. Furthermore, we confirmed that IL‐23 could bind to these receptor heteromers using NanoBRET and phospho‐STAT3 transduction assays. Taken together with the observation that treatment of HiBit‐IL23R:LgBit‐L12Rβ1 or SmBit‐IL23R:LgBit‐L12Rβ1 heteromeric complexes with IL‐23 did not lead to an increase in luminescence, these data indicate that ligand‐induced dimerisation is not the sole factor governing receptor activation.

Whilst we could not completely rule out the possibility that HiBit:LgBit interactions were contributing to complex formation, we found that the affinity of HiBit and LgBit for their corresponding NanoBiT fragment was greatly reduced when fused to IL‐23 receptor monomers. The effect was most marked for LgBit‐IL12Rβ1, which did not generate a saturable luminescence signal with increasing concentrations of purified HiBit over the concentration range tested, having a likely affinity in the order of 100–1000 nM, making this the limiting affinity for NanoBiT complementation driven heteromer formation. These data strongly suggest that the NanoBiT tags are not driving the interactions observed between IL23R and IL12Rβ1.

Utilising a NanoBiT BRET binding assay, in which light generated from complemented receptors was used to excite fluorophore conjugated IL‐23 (IL23‐TMR), it was possible to monitor binding interactions of IL‐23 solely to pre‐formed heteromers. This approach demonstrated an affinity of pre‐formed IL23R‐IL12Rβ1 heteromers for IL23‐TMR similar to that previously measured for IL23‐TMR binding to NanoLuc tagged receptor heteromers. Interestingly, the equilibrium dissociation constant was between that of IL23‐TMR binding to cells expressing NL‐IL23R and IL12Rβ1 and those expressing NL‐IL12Rβ1 and IL23R. The hypothesis previously suggested for the difference in these affinities, was that tagging IL12Rβ1 with NanoLuc led to greater steric interference in the binding of the ligand (Lay et al., 2022). A NanoBiT heteromer binding affinity mid‐way between these values suggests that the position of the complemented luciferase interferes less in binding than NanoLuc fused to IL12Rβ1, but more than NanoLuc fused to IL23R. As the affinity of IL23‐TMR binding to NanoBiT complemented heteromers is equivalent to the affinities measured for the interaction of the probe with NanoLuc‐tagged heteromers, and that IL‐23 does not induce heteromerisation, it is most likely that heteromers are pre‐formed prior to ligand binding in both experiments.

We previously observed that binding of IL‐23 to its receptor leads to a change in the BRET ratio between *N‐*terminal donor and acceptor tags, and hypothesised that this was due to a ligand induced change in the proximity or orientation of the *N‐*terminal domains (Lay et al., 2022). By utilising the NanoBiT system, which is less influenced by orientation than BRET due to the importance of dipole–dipole interactions for resonance energy transfer, we were able to focus primarily on ligand induced changes in the proximity of the *N‐*terminal domains of the receptor (Machleidt et al., 2015). The lack of a change in luminescence when ligand was added to NanoBiT complemented complexes, indicated that IL‐23 induces a change in orientation rather than proximity of the *N‐*terminal domains.

Measurement of basal STAT3 phosphorylation in the absence of ligand stimulation demonstrated that cells transfected with HiBit‐IL23R and LgBit‐IL12Rβ1 had higher levels of phospho‐STAT3 than untagged receptor or SmBit‐IL23R and LgBit‐IL12Rβ1 transfected cells. This observation is most likely due to the *N‐*termini of the receptor monomers being constrained by the NanoBiT complementation in a favourable conformation for ligand independent activation; however, the ability to raise the level of STAT3 phosphorylation further by the introduction of IL‐23 indicates that these complexes are not locked in a fully active conformation. The observation that increasing the proximity of the *N‐*terminal domains by truncating the linker between HiBit and IL23R by 18 amino acids leads to no change in this basal activation confirms that the activation is not directly related to proximity.

It has been shown that both the *N‐*terminal and *C‐*terminal domains of the IL‐23 receptor undergo a shift in position on ligand binding (Lay et al., 2022; Sivanesan et al., 2016). These observations led to the suggestion that the IL‐23 receptor is activated through a ‘scissor‐like’ conformational change in a similar manner to the Erythropoietin receptor in which the *C‐*terminal domains of the receptor are brought into closer proximity allowing trans‐phosphorylation of *C‐*terminally associated JAK proteins (Sivanesan et al., 2016). Our observation of ligand independent signalling when the *N*‐terminal domains of the receptor are constrained in specific orientations is consistent with this hypothesis. It is possible that this finding indicates that the wildtype IL‐23 receptor could be activated by a synthetic bitopic ligand capable of constraining the *N‐*terminal domains of the receptor in an active conformation. Indeed, it has previously been shown that a chimeric IL‐23 receptor, with extracellular domains switched for anti‐mCherry or GFP nanobodies, could be activated by the application of a bitopic ligand consisting of a mCherry‐GFP fusion (Engelowski et al., 2018).

We also examined if heteromers containing more than one IL23R molecule would associate on the cell surface in the absence of ligand. We found that complexes containing HiBit‐IL23R or SmBit‐IL23R and LgBit‐IL23R gave half the signal of complexes containing HiBit or SmBit‐IL23R and LgBit‐IL12Rβ1. We also examined if these homomeric complexes could engage ligand, finding that when co‐expressed with IL12Rβ1, the majority of homomers were disrupted; however, the remaining complexes could engage the IL23‐TMR. As IL23R and IL12Rβ1 must be present to form the high affinity IL‐23 binding site (Lay et al., 2022), a multimeric species consisting of at least two IL23R molecules and IL12Rβ1 must be engaging IL‐23 in this situation. Taken together, our results indicate that multimeric IL‐23 receptor species containing multiple IL23R can be induced to form with no reduction in ligand binding affinity; therefore, the formation of higher order receptor structures, as is the case for other closely related receptors (Ward et al., 1994), cannot be ruled out.

Our finding that the affinity of purified LgBit for HiBit‐IL23R was significantly decreased when co‐expressed with IL12Rβ1 indicates that IL12Rβ1 shields the *N*‐termini of HiBit‐IL23R in the IL‐23 receptor complex. As the affinity of LgBit is reduced to a similar degree both in the presence and absence of IL‐23, it is likely that in both conformations the *N‐*terminal domain of IL23R is in close association with IL12Rβ1.

We also evaluated whether increasing expression of HT‐IL12Rβ1 could influence the formation of SmBit containing NanoBiT heteromers. This demonstrated that HT‐IL12Rβ1 could compete with LgBit‐IL12Rβ1 for SmBit‐IL23R and that this was independent of the presence of ligand, further supporting the ligand induced conformational change hypothesis. This successful disruption of IL‐23 receptor heteromers raises the possibility of the development of peptide blockers of heteromerisation based on the structure of IL12Rβ1. Numerous examples of membrane‐spanning blocking peptides have been reported that act as allosteric inhibitors of complex formation (Stone & Deber, 2017). Such molecules could be useful tools to further study heteromer formation and a potential novel therapeutic tactic to antagonise IL‐23 signalling at the heteromeric interface. A crucial piece of information for this approach is the location of the formation of heteromeric receptor species. In our study, we observed luminescence at intracellular foci and on the cell membrane when imaging cells expressing HiBit‐IL23R and LgBit‐IL12Rβ1. Other cytokine receptors such as the growth hormone receptor have been shown to be exported from the endoplasmic reticulum as a dimer (Gent et al., 2002); however, studies on IL23R have shown that the molecule is internalised after ligand stimulation and recycled to the cell surface in the absence of IL12Rβ1 (Sun et al., 2020).

Whilst single transmembrane receptors were originally thought to be activated through dimerisation, the growing list of receptors that are heteromeric and inactive prior to ligand binding suggests that many receptors may be activated by a conformational rather than oligomeric mechanism, for example, ligand induced rotation (Maruyama, 2015; Westerfield & Barrera, 2020). Our study demonstrates that when co‐expressed on HEK293T cells, IL23R and IL12Rβ1 associate in the absence of ligand to form inactive complexes and are therefore unlikely to be activated by dimerisation alone. The precise mechanism by which these heteromers are activated remains to be elucidated. Whilst the structure of IL‐23 in complex with the purified and truncated IL23R domain 1–3 (Bloch et al., 2018) and more recently with IL12Rβ1 domain 1 (Glassman et al., 2021) have been solved, structural elucidation of the remainder of IL12Rβ1 was not possible, and both studies indicated that the affinity of purified truncates of IL12Rβ1 was much lower for IL‐23 than when the receptor is expressed in the membrane of living cells (Lay et al., 2022). Cryo‐EM of liganded and unliganded receptor complexes solubilised in a lipid membrane would be a useful next step in the defining the conformational change that leads to receptor activation. Structural studies utilising mutagenesis, conformational restraint, crystallography and molecular modelling have been used to investigate the activation mechanisms of related cytokine receptors (Brown et al., 2005; Lu et al., 2006; Poger & Mark, 2010; Seubert et al., 2003). Techniques such as these could be applied to IL‐12 family cytokine receptors to further understand receptor activation.

## 5 CONCLUSION

The IL‐23 cytokine and its receptor are important drug targets for the treatment of auto‐inflammatory conditions. Although receptor antagonists are undergoing clinical trials, anti‐IL‐23 biologics are currently the only licensed therapeutics to specifically target the pathway. To successfully target the receptor, further information is needed on the activation mechanism by which IL‐23 binding translates to signalling. Conflicting hypotheses have been suggested in which the receptor is activated either by ligand induced dimerisation or a ligand induced conformational change. This study demonstrated that inactive complexes of the IL‐23 receptor form prior to cytokine engagement, with the addition of ligand leading to no increase in association between receptor monomers. In addition, we demonstrated that high affinity constraint of the *N‐*terminal domains of the receptor leads to ligand independent activation and that complex formation could be inhibited by increasing concentrations of competing receptor monomers. These results indicate that association of receptor monomers alone is not sufficient to induce signalling and a conformational change of associated heteromeric complexes is likely necessary to enable signal transduction. These observations have direct implications on current efforts to target the IL‐23 pathway by raising the possibility of allosteric modulators capable of disrupting ligand independent heteromer formation or ligand induced re‐arrangement.

## CONFLICT OF INTEREST

P.D.C. is an employee of GlaxoSmithKline.

## AUTHOR CONTRIBUTIONS

**Charles S. Lay:** Conceptualization; data curation; formal analysis; investigation; methodology; writing‐original draft; writing‐review and editing. **Laura E. Kilpatrick:** Investigation; methodology; writing‐original draft; writing‐review and editing. **Peter D. Craggs:** Conceptualization; funding acquisition; project administration; supervision; writing‐original draft; writing‐review and editing. **Stephen John Hill:** Conceptualization; funding acquisition; project administration; resources; supervision; writing‐original draft; writing‐review and editing.

## DECLARATION OF TRANSPARENCY AND SCIENTIFIC RIGOUR

This Declaration acknowledges that this paper adheres to the principles for transparent reporting and scientific rigour of preclinical research as stated in the *BJP* guidelines for Design and Analysis, and as recommended by funding agencies, publishers and other organisations engaged with supporting research.

## Data Availability

The data that support the findings of this study are available from the corresponding author upon reasonable request.

## References

[bph16018-bib-0001] Alexander, S. P. H. , Fabbro, D. , Kelly, E. , Mathie, A. , Peters, J. A. , Veale, E. L. , Armstrong, J. F. , Faccenda, E. , Harding, S. D. , Pawson, A. J. , Southan, C. , Davies, J. A. , Beuve, A. , Brouckaert, P. , Bryant, C. , Burnett, J. C. , Farndale, R. W. , Friebe, A. , Garthwaite, J. , … Waldman, S. A. (2021a). The concise guide to pharmacology 2021/22: Catalytic receptors. British Journal of Pharmacology, 178, S264–S312. 34529829 10.1111/bph.15541

[bph16018-bib-0002] Alexander, S. P. H. , Fabbro, D. , Kelly, E. , Mathie, A. , Peters, J. A. , Veale, E. L. , Armstrong, J. F. , Faccenda, E. , Harding, S. D. , Pawson, A. J. , Southan, C. , Davies, J. A. , Boison, D. , Burns, K. E. , Dessauer, C. , Gertsch, J. , Helsby, N. A. , Izzo, A. A. , Koesling, D. , Ostrom, R. , Pyne, N. J. , Pyne, S. , Russwurm, M. , Seifert, R. , Stasch, J.‐P. , van der Stelt, M. , van der Vliet, A. , Watts, V. & Wong, S. S. (2021b). THE CONCISE GUIDE TO PHARMACOLOGY 2021/22: Enzymes. British Journal of Pharmacology, 178(S1), S313‐S411. 10.1111/bph.15542 34529828

[bph16018-bib-0003] Alexander, S. P. H. , Kelly, E. , Mathie, A. , Peters, J. A. , Veale, E. L. , Armstrong, J. F. , Faccenda, E. , Harding, S. D. , Pawson, A. J. , Southan, C. , Buneman, O. P. , Cidlowski, J. A. , Christopoulos, A. , Davenport, A. P. , CGTP Collaborators , Fabbro, D. , Spedding, M. , Striessnig, J. , Davies, J. A. , … Zolghadri, Y. (2021a). THE CONCISE GUIDE TO PHARMACOLOGY 2021/22: Introduction and Other Protein Targets. British Journal of Pharmacology, 178(S1), S1–S26. 10.1111/bph.15537 34529830 PMC9513948

[bph16018-bib-0004] Bloch, Y. , Bouchareychas, L. , Merceron, R. , Składanowska, K. , Van den Bossche, L. , Detry, S. , Govindarajan, S. , Elewaut, D. , Haerynck, F. , Dullaers, M. , Adamopoulos, I. E. , & Savvides, S. N. (2018). Structural activation of pro‐inflammatory human cytokine IL‐23 by cognate IL‐23 receptor enables recruitment of the shared receptor IL‐12Rβ1. Immunity, 48, 45–58. 10.1016/j.immuni.2017.12.008 29287995 PMC5773378

[bph16018-bib-0005] Brown, R. J. , Adams, J. J. , Pelekanos, R. A. , Wan, Y. , McKinstry, W. J. , Palethorpe, K. , Seeber, R. M. , Monks, T. A. , Eidne, K. A. , Parker, M. W. , & Waters, M. J. (2005). Model for growth hormone receptor activation based on subunit rotation within a receptor dimer. Nature Structural & Molecular Biology, 12, 814–821. 10.1038/nsmb977 16116438

[bph16018-bib-0006] Cheng, X. , Lee, T.‐Y. , Ledet, G. , Zemade, G. , Tovera, J. , Campbell, R. , Purro, N. , Annamalai, T. , Masjedizadeh, M. , Liu, D. , & Nawabi, R. (2019). Safety, tolerability, and pharmacokinetics of PTG‐200, an oral GI‐restricted peptide antagonist of IL‐23 receptor, in normal healthy volunteers. The American Journal of Gastroenterology, 114, 439–440.

[bph16018-bib-0007] Chyuan, I. T. , & Lai, J. H. (2020). New insights into the IL‐12 and IL‐23: From a molecular basis to clinical application in immune‐mediated inflammation and cancers. Biochemical Pharmacology, 175, 1–9. 10.1016/j.bcp.2020.113928 32217101

[bph16018-bib-0008] Curtis, M. J. , Alexander, S. P. H. , Cirino, G. , George, C. H. , Kendall, D. A. , Insel, P. A. , Izzo, A. A. , Ji, Y. , Panettieri, R. A. , Patel, H. H. , Sobey, C. G. , Stanford, S. C. , Stanley, P. , Stefanska, B. , Stephens, G. J. , Teixeira, M. M. , Vergnolle, N. , & Ahluwalia, A. (2022). Planning experiments: Updated guidance on experimental design and analysis and their reporting III. British Journal of Pharmacology, 179, 3907–3913. 10.1111/bph.15868 35673806

[bph16018-bib-0009] Dale, N. C. , Johnstone, E. K. M. , White, C. W. , & Pfleger, K. D. G. (2019). NanoBRET: The bright future of proximity‐based assays. Frontiers in Bioengineering and Biotechnology, 7, 1–13. 30972335 10.3389/fbioe.2019.00056 PMC6443706

[bph16018-bib-0010] Dixon, A. S. , Schwinn, M. K. , Hall, M. P. , Zimmerman, K. , Otto, P. , Lubben, T. H. , Butler, B. L. , Binkowski, B. F. , Machleidt, T. , Kirkland, T. A. , Wood, M. G. , Eggers, C. T. , Encell, L. P. , & Wood, K. V. (2016). NanoLuc complementation reporter optimized for accurate measurement of protein interactions in cells. ACS Chemical Biology, 11, 400–408. 10.1021/acschembio.5b00753 26569370

[bph16018-bib-0011] Engelowski, E. , Schneider, A. , Franke, M. , Xu, H. , Clemen, R. , Lang, A. , Baran, P. , Binsch, C. , Knebel, B. , Al‐Hasani, H. , Moll, J. M. , Floß, D. M. , Lang, P. A. , & Scheller, J. (2018). Synthetic cytokine receptors transmit biological signals using artificial ligands. Nature Communications, 9, 1–15. 10.1038/s41467-018-04454-8 PMC5964073 29789554

[bph16018-bib-0012] Floss, D. M. , Mrotzek, S. , Klöcker, T. , Schröder, J. , Grötzinger, J. , Rose‐John, S. , & Scheller, J. (2013). Identification of canonical tyrosine‐dependent and non‐canonical tyrosine‐independent STAT3 activation sites in the intracellular domain of the interleukin 23 receptor. The Journal of Biological Chemistry, 288, 19386–19400. 10.1074/jbc.M112.432153 23673666 PMC3707643

[bph16018-bib-0013] Gaffen, S. L. , Jain, R. , Garg, A. V. , & Cua, D. J. (2014). The IL‐23‐IL‐17 immune axis: From mechanisms to therapeutic testing. Nature Reviews. Immunology, 14, 585–600. 10.1038/nri3707 PMC4281037 25145755

[bph16018-bib-0014] Gent, J. , Van Kerkhof, P. , Roza, M. , Bu, G. , & Strous, G. J. (2002). Ligand‐independent growth hormone receptor dimerization occurs in the endoplasmic reticulum and is required for ubiquitin system‐dependent endocytosis. Proceedings of the National Academy of Sciences of the United States of America, 99, 9858–9863. 10.1073/pnas.152294299 12105275 PMC125043

[bph16018-bib-0015] Glassman, C. R. , Mathiharan, Y. K. , Jude, K. M. , Su, L. , Panova, O. , Lupardus, P. J. , Spangler, J. B. , Ely, L. K. , Thomas, C. , Skiniotis, G. , & Garcia, K. C. (2021). Structural basis for IL‐12 and IL‐23 receptor sharing reveals a gateway for shaping actions on T versus NK cells. Cell, 184, 983–999. 10.1016/j.cell.2021.01.018 33606986 PMC7899134

[bph16018-bib-0016] Kagami, S. , Rizzo, H. L. , Kurtz, S. E. , Miller, L. S. , & Blauvelt, A. (2010). IL‐23 and IL‐17A, but not IL‐12 and IL‐22, are required for optimal skin host defense against *Candida albicans* . Journal of Immunology, 185, 5453–5462. 10.4049/jimmunol.1001153 PMC3076054 20921529

[bph16018-bib-0017] Lay, C. S. , Bridges, A. , Goulding, J. , Briddon, S. J. , Soloviev, Z. , Craggs, P. D. , & Hill, S. J. (2022). Probing the binding of interleukin‐23 to individual receptor components and the IL‐23 heteromeric receptor complex in living cells using NanoBRET. Cell Chemical Biology, 29, 19–29. 10.1016/j.chembiol.2021.05.002 34038748 PMC8790524

[bph16018-bib-0018] Lu, X. , Gross, A. W. , & Lodish, H. F. (2006). Active conformation of the erythropoietin receptor: Random and cysteine‐scanning mutagenesis of the extracellular juxtamembrane and transmembrane domains. The Journal of Biological Chemistry, 281, 7002–7011. 10.1074/jbc.M512638200 16414957

[bph16018-bib-0019] Luo, X. , Chen, O. , Wang, Z. , Bang, S. , Ji, J. , Lee, S. H. , Huh, Y. , Furutani, K. , He, Q. , Tao, X. , Ko, M. C. , Bortsov, A. , Donnelly, C. R. , Chen, Y. , Nackley, A. , Berta, T. , & Ji, R. R. (2021). IL‐23/IL‐17A/TRPV1 axis produces mechanical pain via macrophage‐sensory neuron crosstalk in female mice. Neuron, 109, 2691–2706. 10.1016/j.neuron.2021.06.015 34473953 PMC8425601

[bph16018-bib-0020] Machleidt, T. , Woodroofe, C. C. , Schwinn, M. K. , Méndez, J. , Robers, M. B. , Zimmerman, K. , Otto, P. , Daniels, D. L. , Kirkland, T. A. , & Wood, K. V. (2015). NanoBRET‐A novel BRET platform for the analysis of protein‐protein interactions. ACS Chemical Biology, 10, 1797–1804. 10.1021/acschembio.5b00143 26006698

[bph16018-bib-0021] Maruyama, I. N. (2015). Activation of transmembrane cell‐surface receptors via a common mechanism? The ‘rotation model’. BioEssays, 37, 959–967. 10.1002/bies.201500041 26241732 PMC5054922

[bph16018-bib-0022] Mirlekar, B. , & Pylayeva‐Gupta, Y. (2021). IL‐12 family cytokines in cancer and immunotherapy. Cancers (Basel)., 13, 167. 10.3390/cancers13020167 33418929 PMC7825035

[bph16018-bib-0023] Oppmann, B. , Lesley, R. , Blom, B. , Timans, J. C. , Xu, Y. , Hunte, B. , Vega, F. , Yu, N. , Wang, J. , Singh, K. , Zonin, F. , Vaisberg, E. , Churakova, T. , Liu, M. R. , Gorman, D. , Wagner, J. , Zurawski, S. , Liu, Y. J. , Abrams, J. S. , … Kastelein, R. A. (2000). Novel p19 protein engages IL‐12p40 to form a cytokine, IL‐23, with biological activities similar as well as distinct from IL‐12. Immunity, 13, 715–725. 10.1016/S1074-7613(00)00070-4 11114383

[bph16018-bib-0024] Parham, C. , Chirica, M. , Timans, J. , Vaisberg, E. , Travis, M. , Cheung, J. , Pflanz, S. , Zhang, R. , Singh, K. P. , Vega, F. , To, W. , Wagner, J. , O'Farrell, A. M. , McClanahan, T. , Zurawski, S. , Hannum, C. , Gorman, D. , Rennick, D. M. , Kastelein, R. A. , … Moore, K. W. (2002). A receptor for the heterodimeric cytokine IL‐23 is composed of IL‐12Rβ1 and a novel cytokine receptor subunit, IL‐23R. Journal of Immunology, 168, 5699–5708. 10.4049/jimmunol.168.11.5699 12023369

[bph16018-bib-0041] Peach, C. J. , Kilpatrick, L. E. , Woolard, J. , & Hill, S. J. (2021). Use of NanoBiT and NanoBRET to monitor fluorescent VEGF‐A binding kinetics to VEGFR2/NRP1 heteromeric complexes in living cells. British Journal of Pharmacology, 178(12), 2393–2411. 10.1111/bph.15426 33655497

[bph16018-bib-0025] Poger, D. , & Mark, A. E. (2010). Turning the growth hormone receptor on: Evidence that hormone binding induces subunit rotation. Proteins Struct. Funct. Bioinforma., 78, 1163–1174. 10.1002/prot.22636 19927328

[bph16018-bib-0026] Schroder, J. , Moll, J. M. , Baran, P. , Grotzinger, J. , Scheller, J. , & Floss, D. M. (2015). Non‐canonical interleukin 23 receptor complex assembly: P40 protein recruits interleukin 12 receptor β1 via site II and induces P19/interleukin 23 receptor interaction via site III. The Journal of Biological Chemistry, 290, 359–370. 10.1074/jbc.M114.617597 25371211 PMC4281739

[bph16018-bib-0027] Seubert, N. , Royer, Y. , Staerk, J. , Kubatzky, K. F. , Moucadel, V. , Krishnakumar, S. , Smith, S. O. , & Constantinescu, S. N. (2003). Active and inactive orientations of the transmembrane and cytosolic domains of the erythropoietin receptor dimer. Molecular Cell, 12, 1239–1250. 10.1016/S1097-2765(03)00389-7 14636581

[bph16018-bib-0028] Sivanesan, D. , Beauchamp, C. , Quinou, C. , Lee, J. , Lesage, S. , Chemtob, S. , Rioux, J. D. , & Michnick, S. W. (2016). IL23R (interleukin 23 receptor) variants protective against inflammatory bowel diseases (IBD) display loss of function due to impaired protein stability and intracellular trafficking. The Journal of Biological Chemistry, 291, 8673–8685. 10.1074/jbc.M116.715870 26887945 PMC4861437

[bph16018-bib-0029] Soave, M. , Heukers, R. , Kellam, B. , Woolard, J. , Smit, M. J. , Briddon, S. J. , & Hill, S. J. (2020). Monitoring allosteric interactions with CXCR4 using NanoBiT conjugated nanobodies. Cell Chemical Biology, 27, 1250–1261. 10.1016/j.chembiol.2020.06.006 32610042 PMC7573392

[bph16018-bib-0030] Soave, M. , Kellam, B. , Woolard, J. , Briddon, S. J. , & Hill, S. J. (2020). NanoBiT complementation to monitor agonist‐induced adenosine A1 receptor internalization. SLAS Discov., 25, 186–194. 10.1177/2472555219880475 31583945 PMC6974774

[bph16018-bib-0031] Stone, T. A. , & Deber, C. M. (2017). Therapeutic design of peptide modulators of protein‐protein interactions in membranes. Biochim. Biophys. Acta ‐ Biomembr., 1859, 577–585. 10.1016/j.bbamem.2016.08.013 27580024

[bph16018-bib-0032] Sun, R. , Hedl, M. , & Abraham, C. (2020). IL23R recycling and amplifies innate receptor‐induced signalling and cytokines in human macrophages, and the IBD‐protective IL23R R381Q variant modulates these outcomes. Gut, 69, 264–273. 10.1136/gutjnl-2018-316830 31097538 PMC6858485

[bph16018-bib-0033] Tait Wojno, E. D. , Hunter, C. A. , & Stumhofer, J. S. (2019). The immunobiology of the Interleukin‐12 family: Room for discovery. Immunity, 50, 851–870. 10.1016/j.immuni.2019.03.011 30995503 PMC6472917

[bph16018-bib-0034] Tang, C. , Chen, S. , Qian, H. , & Huang, W. (2012). Interleukin‐23: As a drug target for autoimmune inflammatory diseases. Immunology, 135, 112–124. 10.1111/j.1365-2567.2011.03522.x 22044352 PMC3277713

[bph16018-bib-0035] Verreck, F. A. W. , De Boer, T. , Langenberg, D. M. L. , Hoeve, M. A. , Kramer, M. , Vaisberg, E. , Kastelein, R. , Kolk, A. , de Waal‐Malefyt, R. , & Ottenhoff, T. H. (2004). Human IL‐23‐producing type 1 macrophages promote but IL‐10‐producing type 2 macrophages subvert immunity to (myco)bacteria. Proceedings of the National Academy of Sciences of the United States of America, 101, 4560–4565. 10.1073/pnas.0400983101 15070757 PMC384786

[bph16018-bib-0036] Wang, X. , Wei, Y. , Xiao, H. , Liu, X. , Zhang, Y. , Han, G. , Chen, G. , Hou, C. , Ma, N. , Shen, B. , Li, Y. , Egwuagu, C. E. , & Wang, R. (2016). A novel IL‐23p19/Ebi3 (IL‐39) cytokine mediates inflammation in lupus‐like mice. European Journal of Immunology, 46, 1343–1350. 10.1002/eji.201546095 27019190 PMC11334612

[bph16018-bib-0037] Ward, L. D. , Howlett, G. J. , Discolo, G. , Yasukawa, K. , Hammacher, A. , Moritz, R. L. , & Simpson, R. J. (1994). High affinity interleukin‐6 receptor is a hexameric complex consisting of two molecules each of interleukin‐6, interleukin‐6 receptor, and gp‐130. The Journal of Biological Chemistry, 269, 23286–23289. 10.1016/S0021-9258(17)31651-4 8083235

[bph16018-bib-0038] Westerfield, J. M. , & Barrera, F. N. (2020). Membrane receptor activation mechanisms and transmembrane peptide tools to elucidate them. The Journal of Biological Chemistry, 295, 1792–1814. 10.1074/jbc.REV119.009457 31879273 PMC7029107

[bph16018-bib-0039] Ye, J. , Wang, Y. , Wang, Z. , Liu, L. , Yang, Z. , Wang, M. , Xu, Y. , Ye, D. , Zhang, J. , Lin, Y. , Ji, Q. , & Wan, J. (2020). Roles and mechanisms of Interleukin‐12 family members in cardiovascular diseases: Opportunities and challenges. Frontiers in Pharmacology, 11, 129. 10.3389/fphar.2020.00129 32194399 PMC7064549

